# Optical coherence tomography angiography in septic shock: a new frontier in microcirculation assessment

**DOI:** 10.62675/2965-2774.20250070

**Published:** 2025-07-31

**Authors:** André Rosa Alexandre, Ana Teresa Leitão, Pedro Póvoa

**Affiliations:** 1 Hospital da Luz Lisboa Intensive Care Department Lisbon Portugal Intensive Care Department, Hospital da Luz Lisboa, Luz Saúde - Lisbon, Portugal.; 2 NOVA University of Lisbon NOVA Medical School Lisbon Portugal NOVA Medical School, NOVA University of Lisbon - Lisbon Portugal.; 3 Odense University Hospital Center for Clinical Epidemiology and Research Unit of Clinical Epidemiology Odense Denmark Center for Clinical Epidemiology and Research Unit of Clinical Epidemiology, Odense University Hospital - Odense, Denmark.; 4 Hospital de São Francisco Xavier Unidade Local de Saúde Lisboa Ocidental Department of Intensive Care Lisbon Portugal Department of Intensive Care, Hospital de São Francisco Xavier, Unidade Local de Saúde Lisboa Ocidental - Lisbon, Portugal.

## INTRODUCTION

Sepsis is a life-threatening organ dysfunction caused by a dysregulated host response to infection. Septic shock, its most severe form, remains a leading cause of mortality in critically ill patients worldwide.^(1,2)^ It is characterized by profound circulatory, cellular, and metabolic abnormalities, and its management requires early identification and interventions to prevent multi-organ dysfunction.^(3)^ Microcirculatory dysfunction is common in septic shock and is associated with increased morbidity and mortality. This phenomenon involves impaired blood flow distribution at the microvascular level, leading to tissue hypoxia and organ failure.^(4,5)^ Despite its critical role, tools for bedside microcirculation monitoring are limited.^(6)^

Optical coherence tomography angiography (OCTA), a novel imaging modality, offers unique potential for non-invasive, real-time microcirculation assessment, particularly at the retinal level.^(7,8)^ This viewpoint explores the promise of OCTA as a transformative tool in septic shock management.

## MICROCIRCULATORY DYSFUNCTION IN SEPTIC SHOCK

The microcirculation is responsible for oxygen delivery, carbon dioxide uptake, and nutrient exchange at the tissue level. In septic shock, microcirculatory dysfunction is characterized by decreased capillary density, heterogeneous perfusion, and increased vascular permeability. This creates a mismatch between oxygen delivery and consumption, perpetuating cellular injury and organ failure.^(9,10)^

Sublingual handheld vital microscopes, the gold standard for assessing microcirculatory dysfunction, have provided valuable insights. However, these techniques face significant limitations, including operator dependence, inconsistent image quality, and limited applicability in non-sedated or spontaneously breathing patients. Consequently, there is an urgent demand for innovative approaches to address these challenges effectively.^(6,11)^

## OPTICAL COHERENCE TOMOGRAPHY ANGIOGRAPHY: PRINCIPLES AND ADVANTAGES

**Figure 1:**
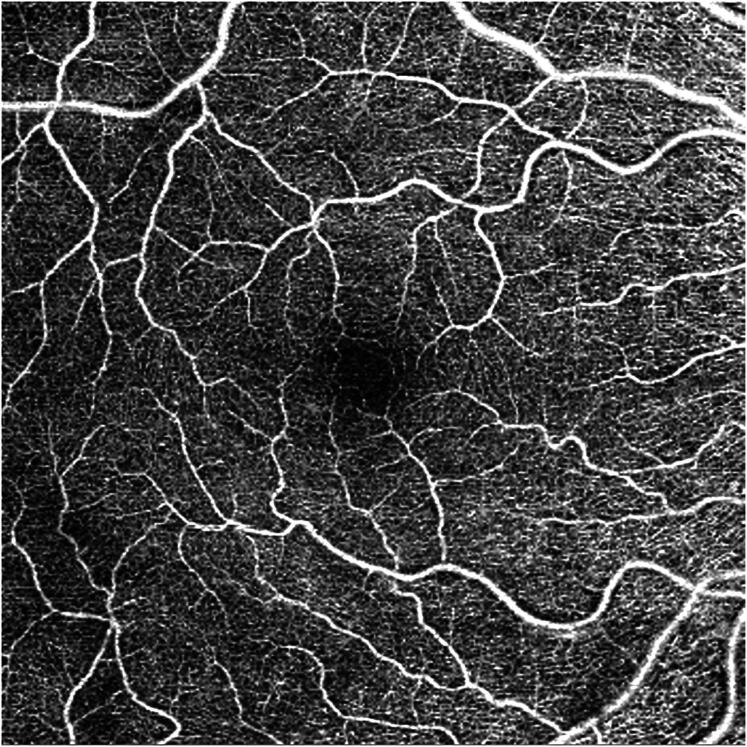
Optical coherence tomography angiography of a septic shock patient showing retinal microvasculature around the foveal avascular zone (middle dark zone).

Optical coherence tomography angiography is an advanced imaging technology that captures detailed images of the retinal microcirculation across multiple layers (Figure 1). It identifies retinal vessels by detecting variations in intensity and/or phase properties of optical coherence tomography signals from red blood cell movement in multiple B-scans. Motion contrast imaging generates angiographic pictures with volumetric blood flow information.^(12)^ Unlike traditional angiographic techniques, OCTA is non-invasive and safe for repeated use, as it does not require contrast agents. Automated image acquisition and interpretation enhance usability by enabling rapid bedside assessment and minimizing operator variability.

Moreover, the retinal microcirculation may better reflect changes in other critical microvascular beds, such as the brain, heart and kidneys, than the sublingual region. The shared embryological origin and similar vascular architecture of retinal and cerebral microcirculation support its use as a window to systemic microcirculation.^(13)^

By leveraging OCTA, clinicians can gain detailed insights into the microvascular changes associated with septic shock, potentially enhancing diagnostic and therapeutic precision.

## POTENTIAL APPLICATION OF OPTICAL COHERENCE TOMOGRAPHY ANGIOGRAPHY IN SEPTIC SHOCK

### Early detection of microcirculatory dysfunction

A significant challenge in septic shock management is the early identification of microcirculatory dysfunction. Macrohemodynamic parameters, such as mean arterial pressure or cardiac output, inadequately reflect microvascular perfusion.^(14)^ Optical coherence tomography angiography provides direct visualization of the retinal microvasculature, enabling the detection of early signs of capillary rarefaction, reduced perfusion density, or vascular leakage.

### Monitoring therapeutic interventions

Optical coherence tomography angiography can potentially monitor the effects of therapeutic intervention in septic shock. Treatments such as fluid resuscitation, vasopressor, or inotropic therapy aim to optimize perfusion and oxygen delivery. By assessing changes in retinal microcirculatory metrics before and after these interventions, OCTA could help guide personalized treatment strategies. For example, increased capillary density on OCTA may indicate improved microvascular perfusion and therapeutic response.^(13)^ In the future, OCTA could be integrated into global treatment algorithms addressing macro- and micro-hemodynamic resuscitation.

### Prognostication and risk stratification

Microcirculatory dysfunction correlates with worse outcomes in septic shock.^(14)^ Optical coherence tomography angiography could provide prognostic information by identifying patients with microcirculatory impairment who are at increased risk of organ failure and death. Parameters such as foveal avascular zone area and perimeter or vessel and perfusion density could be used to stratify patients and tailor treatment intensity. Furthermore, longitudinal monitoring with OCTA could help track disease progression and recovery.

## LIMITATIONS AND CHALLENGES

Although OCTA offers several advantages, its implementation in the intensive care unit (ICU) is challenging.

First, current OCTA devices are relatively bulky and lack portability. However, recent innovations in portable probes and miniaturized devices promise to overcome this barrier.^(7,15)^

Second, interpreting OCTA images requires familiarity with the technology and its outputs. Although automated software simplifies image analysis, training ICU teams to integrate OCTA into routine practice remains essential. Multidisciplinary collaboration between intensivists, ophthalmologists, and biomedical engineers could facilitate this process.

Finally, cost considerations may limit the widespread adoption of OCTA in ICUs. While more affordable than traditional angiography systems, integrating OCTA into resource-limited settings may require careful planning and investment.

## FUTURE DIRECTIONS

To fully explore the potential of OCTA in septic shock, several research avenues warrant exploration:

- **Validation studies:** our group's recent preliminary report is the first to demonstrate OCTA's feasibility for detecting retinal microcirculatory dysfunction in septic shock.^(15)^ Large-scale, multicenter studies are needed to validate OCTA-derived metrics as reliable indicators of microcirculatory dysfunction.
- **Technological innovations:** ongoing innovation in device portability and image analysis algorithms will enhance the feasibility of OCTA in ICU settings.
- **Comparative studies:** head-to-head comparisons between OCTA and existing modalities like handheld vital microscopes could provide valuable insights into their relative strengths and limitations.
- **Integration with Artificial Intelligence:** the development of machine learning algorithms could improve the accuracy and efficiency of OCTA image interpretation, enabling real-time decision support for clinicians.
- **Exploration of other vascular beds:** OCTA could be adapted to assess microvascular changes in other accessible tissues, such as conjunctiva or nail fold capillaries.

In summary, OCTA addresses several limitations of current modalities for monitoring microcirculatory dysfunction in septic shock. Its advantages promise to enable early detection, therapeutic monitoring, and prognostication, with potential to transform septic shock management and improve patient outcomes. Further research and technological innovations are needed to overcome current challenges and solidify OCTA's role as a cornerstone of precision medicine in the ICU.
